# Community Transmission of SARS-CoV-2 Omicron Variant, South Korea, 2021

**DOI:** 10.3201/eid2804.220006

**Published:** 2022-04

**Authors:** Eun-Young Kim, Young June Choe, Hanul Park, Hyoseon Jeong, Jae-Hwa Chung, Jeonghee Yu, Hwa-Pyeong Ko, Hyun Jeong Ahn, Mi-Young Go, Ju-Hyung Lee, Won Ick Kim, Bu Sim Lee, Sooyeon Kim, Mi Yu, Jia Kim, Hye Ryeon Lee, Eun Jung Jang, Ji Joo Lee, Hye Young Lee, Jong Mu Kim, Ji Hyun Choi, Sang Eun Lee, Il-Hwan Kim, Ae Kyung Park, Jee Eun Rhee, Eun-Jin Kim, Sangwon Lee, Young-Joon Park

**Affiliations:** Honam Regional Center for Disease Control and Prevention, Gwangju, South Korea (E.-Y. Kim, H. Jeong, J.-H. Chung, J. Yu); Korea University Anam Hospital, Seoul, South Korea (Y.J. Choe);; Korea Disease Control and Prevention Agency, Cheongju, South Korea (H. Park, M. Yu, J. Kim, H.R. Lee, E.J. Jang, J.J. Lee, H.Y. Lee, J.M. Kim, J.H. Choi, S.E. Lee, I.-.H. Kim, A.K. Park, J.E. Rhee, E.-J. Kim, S. Lee, Y.-J. Park);; Gwangju Metropolitan Government, Gwangju (H.-P. Ko);; Jeollabuk-do Government, Jeonju, South Korea (H.J. Ahn, M.-Y. Go);; Jeollabuk-do Center for Infectious Disease Control and Prevention, Jeonju, South Korea (J.-H. Lee);; Jeollanam-do Government, Muan, South Korea (W.I. Kim, B.S. Lee);; Jeollanam-do Communicable Disease Management Support Team, Muan (S. Kim)

**Keywords:** COVID-19, 2019 novel coronavirus disease, coronavirus disease, severe acute respiratory syndrome coronavirus 2, SARS-CoV-2, viruses, respiratory infections, zoonoses, omicron, variant of concern, VOC, South Korea

## Abstract

In South Korea, a November 2021 outbreak caused by severe acute respiratory syndrome coronavirus 2 Omicron variant originated from 1 person with an imported case and spread to households, kindergartens, workplaces, restaurants, and hospitals, resulting in 11 clusters within 3 weeks. An epidemiologic curve indicated rapid community transmission of the Omicron variant.

The severe acute respiratory syndrome coronavirus 2 (SARS-CoV-2) B.1.1.529 (Omicron) variant of concern has been suggested to be more transmissible than previous variants of concern (*1*). We describe an outbreak caused by the Omicron variant that originated from 1 person with an imported case and rapidly spread within 3 weeks to the community in South Korea.

Details of the surveillance and quarantine system in South Korea have been described (*2*). Public health officers interviewed case-patients, and to identify links between clusters, we created epidemic curves and transmission chains according to circumstances and dates of exposure. 

On November 25, 2021, an asymptomatic 32-year-old man who had arrived at Incheon Airport, Seoul, South Korea, from Tehran, Iran, was quarantined in a relative's house; 9 days later (December 5), he tested positive for SARS-CoV-2 (Figure). After contact tracing, household members were confirmed to have SARS-CoV-2 infection, and further transmission to the kindergarten, workplaces, and restaurants was identified (Figure). 

**Figure:**
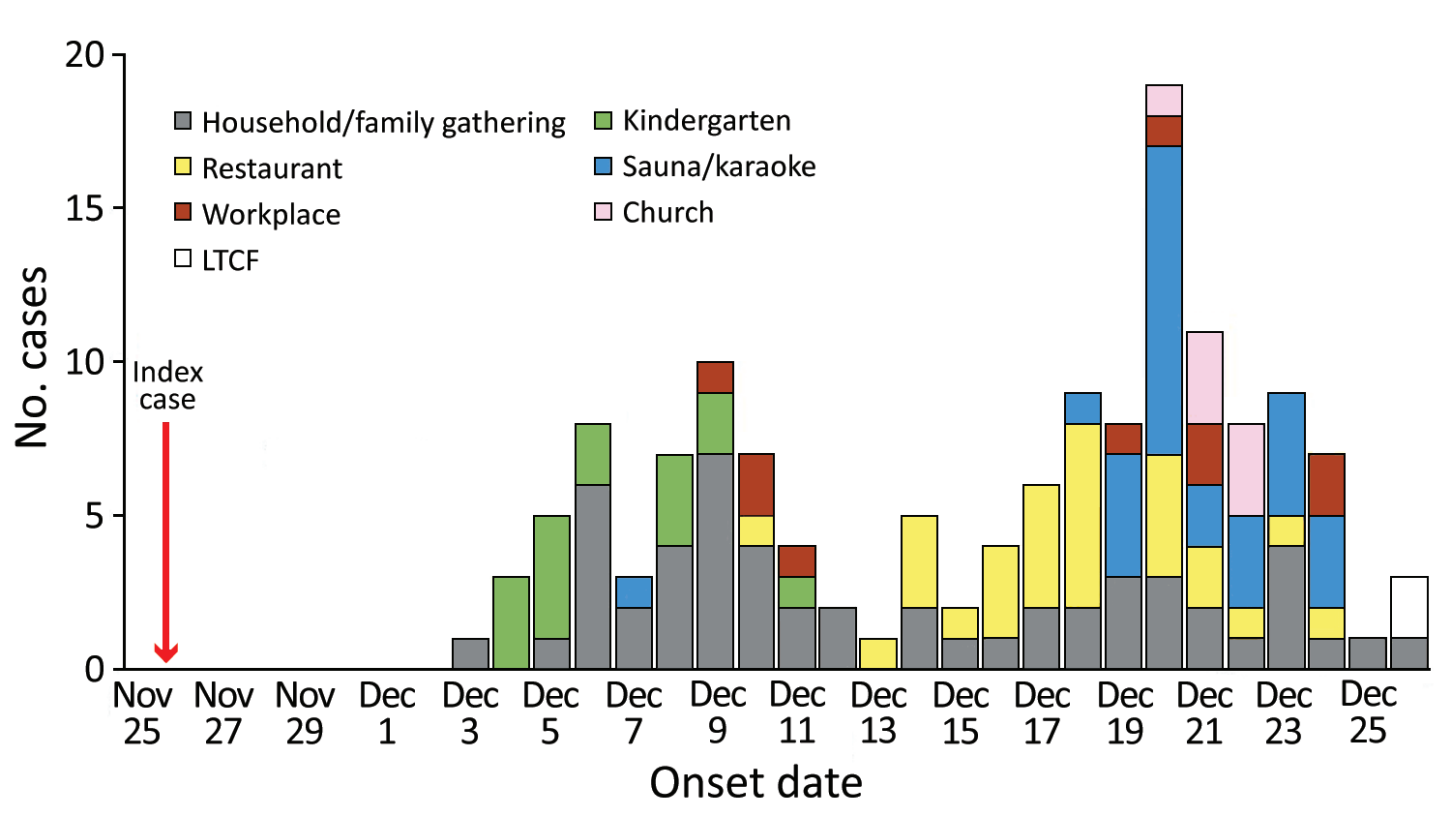
Epidemiologic curve of severe acute respiratory syndrome coronavirus 2 Omicron VOC cluster case-patients, South Korea, 2021. LTCF, long-term care facility.

We confirmed all SARS-CoV-2 cases by using reverse transcription PCR of nasopharyngeal swab specimens. We extracted RNA from the specimens by using a QIAamp Viral RNA Mini Kit (QIAGEN, https://www.qiagen.com), then amplified the receptor-binding domain of the SARS-CoV-2 spike gene by using a One-Step RT-PCR (QIAGEN) with 2 primers selected from ARTIC nCoV-2019 V3 sequencing primer set (https://artic.network/ncov-2019; nCoV-2019_76_LEFT: 5′-AGGGCAAACTGGAAAGATTGCT-3′, nCoV-2019_76_RIGHT 5′-ACACCTGTGCCTGTTAAACCAT-3′). Sequencing of the amplified 417-bp fragments of PCR products (420–543 residues of spike protein) confirmed that the specimens were the Omicron variant. We selected 15 specimens for whole-genome sequencing (WGS) with a QIAGEN QIAseq SARS-CoV-2 Primer Panel and a QIAseq FX DNA Library Kit UDI 1–4 and used NextSeq 1000/2000 P2 Reagents Kit version 3 (Illumina, https://www.illumina.com) for sequencing. For phylogenetic analysis, we aligned SARS-CoV-2 sequences with MAFFT version 7 (*3*) and inferred maximum-likelihood phylogenetic trees with IQTree version 2.1.3 (*4*).

We identified 586 contacts from 29 household clusters, 9 restaurant clusters, 4 workplace clusters, 2 kindergarten clusters, 2 sauna clusters, 2 long-term care facility clusters, 1 karaoke cluster, and 1 church cluster (Appendix Figure 1). A total of 182 of these contacts were verified as case-patients (Table). Community transmission started in the kindergarten and then spread to the workplace, restaurants, sauna/karaoke, long-term care facility, and church (Appendix Figure 1). The secondary attack rates for each cluster were as follows: family gathering, 83.3%; church, 80%; households, 58.9%; restaurants, 46.8%; kindergarten 1, 39.2%; and kindergarten, 2, 24.0% (Appendix Figure 1). As of January 3, 2022, no case-patient was classified as having critical illness or died (Table). WGS showed that virus from 15 household and kindergarten case-patients were closely related to each other and grouped into the same genetic cluster (Table; Appendix Figure 2).

**Table:** Epidemiologic characteristics of 182 severe acute respiratory syndrome coronavirus 2 Omicron variant cluster case-patients, South Korea, November 26–December 26, 2021

Variable	No. (%)
Age group, y	
0–17	32 (17.6)
18–39	52 (28.6)
40–64	70 (38.5)
>65	28 (15.4)
Sex	
F	97 (53.3)
M	85 (46.7)
Transmission site*	
Household	61 (33.7)
Sauna/karaoke	39 (21.5)
Restaurant	30 (16.6)
Kindergarten	19 (10.5)
Workplace	12 (6.6)
Church	8 (4.4)
Long-term care facility	7 (3.9)
Family gathering	5 (2.8)
Vaccination status	
Unvaccinated	3 (20.9)
Partially vaccinated	3 (1.6)
Fully vaccinated	141 (77.5)
Outcome†	
Asymptomatic	39 (21.4)
Critical illness	0
Death	0

*Excluding index case-patient. †As of January 3, 2022.

This outbreak, which was caused by a single-case importation of SARS-CoV-2 Omicron variant to South Korea, started with household transmission to kindergarten and led to 182 cases within 3 weeks, despite high rates of vaccination coverage among adults. As of January 3, 2022, the rate of vaccine coverage in all populations was 83.0% (*5*). Emerging evidence suggests that transmissibility of SARS-CoV-2 Omicron is higher than that for other variants of concern (*1*,*6*). Unlike the previous introduction of the SARS-CoV-2 original strain and variants, Omicron affected children attending kindergarten during its early phase, which partly reflects the immune gap in children. Moreover, the early clusters include family gatherings, restaurants, karaoke events, and saunas, where the universal all-time mask policy may not be feasible, as highlighted in previous studies (*7*). Multifaceted preventive strategies, including vaccination, increasing ventilation, quarantine, and isolation, need to be strengthened to mitigate transmission of the SARS-CoV-2 Omicron variant.

This study is limited because WGS confirmation of the Omicron variant was conducted for selected clusters only and identification of other major clusters was based on field epidemiologic investigations. However, given the thorough contact tracing of the exposed case-patients, all clusters are deemed epidemiologically linked to the Omicron outbreak.

This outbreak demonstrates that despite high vaccination coverage, transmission of the SARS-CoV-2 Omicron variant via symptomatic and asymptomatic persons was rapid, causing community transmission from 1 person with an imported case. As the Omicron variant continues to spread, we suggest vigilant monitoring of childcare facilities and vaccinating of elderly persons with booster doses.

Appendix Supplemental results from study of community transmission of SARS-CoV-2 Omicron variant, South Korea.
