# Supplementary material for: Community Transmission of SARS-CoV-2 Omicron Variant, South Korea, 2021
Source: Emerg Infect Dis. 2022 Apr;28(4):898–900. doi: 10.3201/eid2804.220006 (PMC8962914; doi:10.3201/eid2804.220006)
Supplement: Appendix — Supplemental results from study of community transmission of SARS-CoV-2 Omicron variant, South Korea. [file 22-0006-Techapp-s1.pdf]

Article DOI: https://doi.org/10.3201/eid2804.220006

Community Transmission of SARS-CoV-2 Omicron Variant, South Korea, 2021

Appendix

Appendix Table. Isolation and identification of SARS-CoV-2 virus names, and GISAID accession numbers*

No.	Virus name	Accession no.	Cluster
1	hCoV-19/South Korea/KDCA25927/2021	EPI_ISL_8617644	Kindergarten 1
2	hCoV-19/South Korea/KDCA25922/2021	EPI_ISL_8617638	Family 1
3	In registration process	N/A	Family 1
4	hCoV-19/South Korea/KDCA25933/2021	EPI_ISL_8617650	Kindergarten 1
5	hCoV-19/South Korea/KDCA23400/2021	EPI_ISL_8192593	Kindergarten 2
6	hCoV-19/South Korea/KDCA25928/2021	EPI_ISL_8617645	Kindergarten 2
7	hCoV-19/South Korea/KDCA25925/2021	EPI_ISL_8617641	Kindergarten 2
8	hCoV-19/South Korea/KDCA23395/2021	EPI_ISL_8192588	Kindergarten 2
9	hCoV-19/South Korea/KDCA25924/2021	EPI_ISL_8617640	Family 1
10	hCoV-19/South Korea/KDCA25930/2021	EPI_ISL_8617647	Kindergarten 2
11	hCoV-19/South Korea/KDCA25926/2021	EPI_ISL_8617642	Kindergarten 1
12	hCoV-19/South Korea/KDCA25929/2021	EPI_ISL_8617646	Kindergarten 2
13	hCoV-19/South Korea/KDCA25931/2021	EPI_ISL_8617648	Kindergarten 1
14	hCoV-19/South Korea/KDCA25932/2021	EPI_ISL_8617649	Kindergarten 1
15	hCoV-19/South Korea/KDCA25923/2021	EPI_ISL_8617639	Family 1

*GISAID, http://www.Gisaid.org/database.

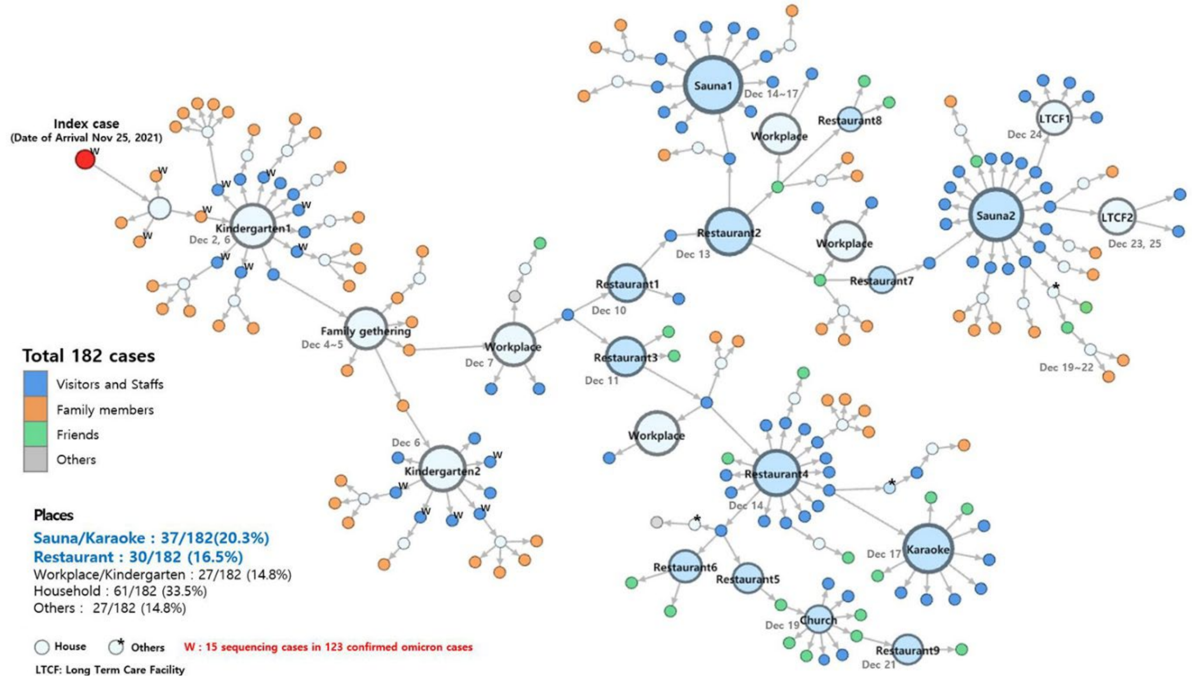

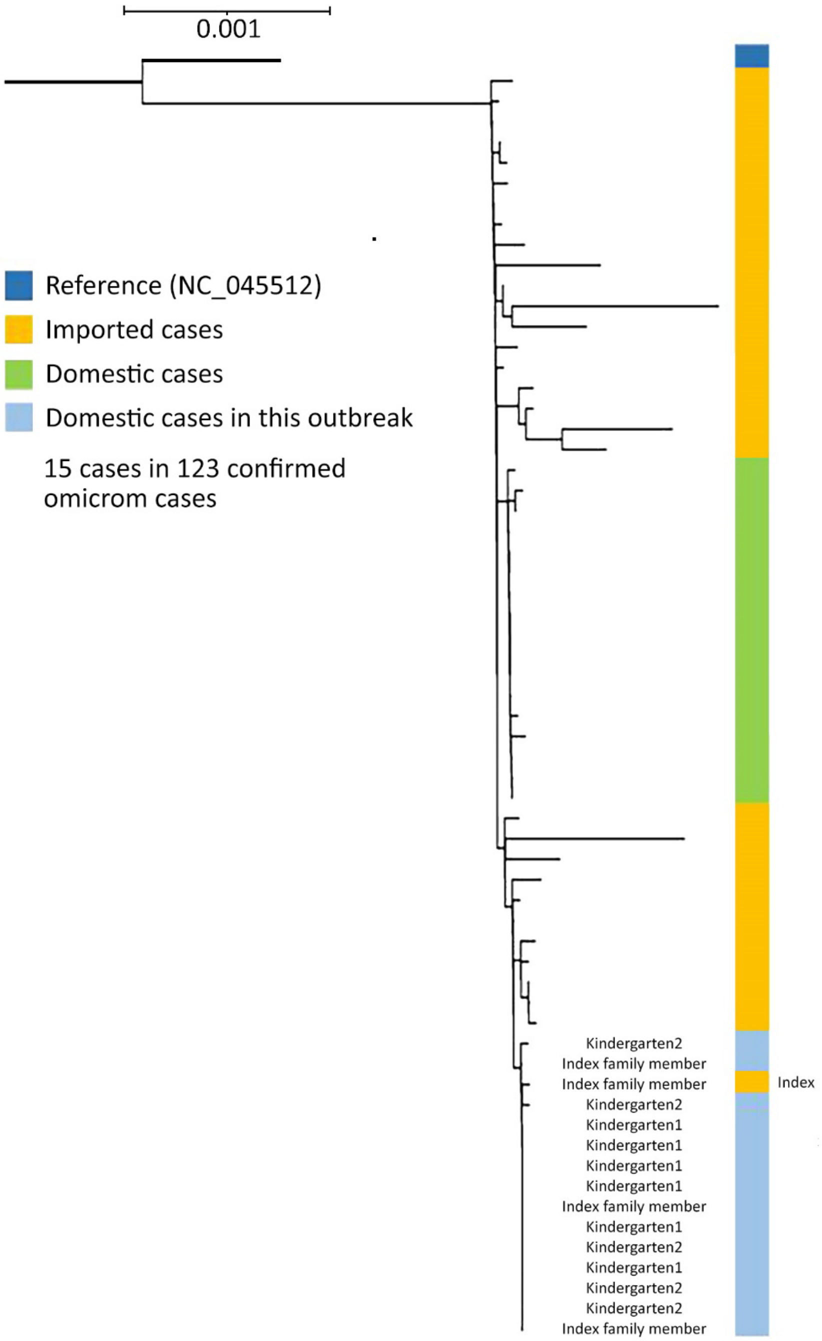

Appendix Figure 2. Phylogenetic analysis of SARS-CoV-2 genome sequences of 15 cases. *The total 15 cases of household member and kindergarten was analyzed with other omicrons in South Korea. These cases were closely related and grouped as a same genetic cluster. The maximum likelihood phylogenetic tree was constructed using with IQTree v.2.1.3. The SARS-CoV-2 whole genome sequences were shared to the GISAID EpiCoV database (http://www.Gisaid.org/database).
